# News and Coming Events

**Published:** 1907-04-27

**Authors:** 


### NEWS AND COMING EVENTS.

Earl Cawdor has presented a complete *x*-ray installation to the Nairn Town and County Hospital.

The Normans Riding Isolation Hospital near Winlayton, completed at a cost of over £6,000, was opened last week.

Mr. Stephen Mayou, F.R.C.S., has been appointed assistant surgeon to the Central London Ophthalmic Hospital, Gray's Inn Road, W.C.

A cot endowed in memory of the late Mr. Roland Philipson has been presented to Newcastle Royal Infirmary by the workmen and apprentices of the North-Eastern Marine Engineering Co., Ltd., Wallsend.

A new Infectious Diseases Hospital has been erected at Caerau, Glamorganshire, the architect being Mr. John H. James, of Cardiff. The sanitary arrangements include the "Boyle" system of natural ventilating.

Dr. D. Davies, who has just resigned the post of medical officer of health for Aberdare, began his connection with the Aberdare Urban District Council as far back as 1854. He was born in 1821, and was one of the "foundation members" of the British Medical Association.

No fewer than 128 competitors entered for the billiard tournament in aid of the Haggerston Hospital Society, and a series of interesting matches has resulted in Mr. R. Robinson winning the first prize, the value of which he has now generously promised to hand over to the funds of the charity. He also presented the runners-up in the competition with miniature silver cups as souvenirs of the event.

Hill's Plymouth (Merthyr) Colliery workmen have decided, by a majority of 250 votes, not to pay an increased subscription annually to the local hospital. When the hospital was extended by two new wards, the representatives of the workmen gave a distinct undertaking that the men would pay an increased subscription. That undertaking was honourably carried out until the Cyfarthfa colliers refused last year to make any increase. The probable result will be a reduction in the number of beds.

A correspondent in *Country Life* reports that at the Royal Hospital for Incurables, Putney Heath, is a fox-terrier bitch whose kennel is near to the gardener's cottage. "Not far away is the poultry-house, where suitable and adequate nesting accommodation is provided. Nothwithstanding this, several of the hens make a practice of going into the terrier's kennel and there depositing eggs. In order to get to the back of the kennel some of the hens will actually walk over the body of the dog, who apparently allows them to do anything, with the exception of tasting her dinner!"

On Friday afternoon last Mr. F. Witherington gave a demonstration of "a new process of shaving without the use of the razor." The demonstration, which was exceedingly interesting, occupied about three quarters of an hour, during which time half a dozen faces were shaved by the new method. This consists simply in painting the beard with the special preparation, keeping it moist with a water spray for a couple of minutes, and then removing the hair with a blunt paper knife. The process, of which we hope to give a fuller account in a later issue, is exceedingly simple, and should prove a boon to those who find the ordinary methods of shaving too tedious and too troublesome.

The foundation-stone of a new hospital at Tullow, co. Callow, Ireland, was laid on Friday last by the Bishop of Kildare.

Lord Loreburn presides at the Westminster Hospital Festival Dinner, to be held in the Hall of the Inner Temple on Friday, May 10, at 8 p.m.

There are only six endowed beds in the Bristol General Hospital. This old institution is finding its accommodation much too small, and is building a new isolation wing at a cost of £12,000.

The Earl Cawdor, as Treasurer of the London Homœopathic Hospital, Great Ormond Street, W.C., has received a legacy of £2,027 4s. 2d. from the estate of the late Mr. William Bykur, of Poole, Dorset.

The annual meeting of the Hospital for Epilepsy and Paralysis, Maida Vale, W., will be held on Saturday, April 27, at 3 p.m. at the hospital, 4 Maida Vale (near the end of St. John's Wood Road). A presentation will be made to the Secretary, Mr. Howgrave Graham, on his retirement after twenty-four years of service.

Following up the suggestion that different professions and trades might endow beds at the Leeds Consumptive Sanatoria for the use of their members, Mr. Irwin Sawdon, the conductor of the Sunday Symphony Society's orchestra, which consists of a chosen body of about fifty professional musicians of Leeds, will give a series of Sunday concerts in aid of this very deserving cause.

The annual Spital Sermon at Newgate on behalf of the ancient, royal, and religious hospitals of London, was this year preached by the Bishop of Southwark. The Spital service is an imposing ceremony, attended by the City Corporation in state, but it is usually a small audience that listens to the sermon. It takes place at three o'clock in the afternoon, and was originally instituted for the benefit of five hospitals only—St. Thomas's, Bethlem, Bridewell, St. Bartholomew's, and Christ's Hospital.

The Bristol branch of the National League of the Blind calls attention to the large number of adult blind whose condition is described as unsatisfactory, and states that the problem can never be solved by voluntary effort. At present there are about 40,000 blind persons in the United Kingdom, and 20,000 blind adults depend on their own efforts for a livelihood. Of these not more than 15 per cent. are employed in workshops, at an average wage of 7s. 1d. per week, and about 50 per cent. subsist upon the rates, whilst 30 per cent. are "forced into the streets" as musicians or beggars.

The following amounts have been promised towards the joint endowment for the Hartlepools hospitals:—Messrs. W. Gray and Co., Limited, £2,000; Sir Christopher Furness, £1,000; Mrs. Matthew Gray, £1,000; Mr. G. H. Bains, £1,000; Messrs. Furness, Withy, and Co., £1,000; Messrs. Richardson, Westgarth and Co., £500; South Durham Steel and Iron Co., £500; the Irvine Shipbuilding and Dry Dock Co., Limited, £500; the North-Eastern Railway Co., £200; Hartlepool Amateur Operative Society, £80; Anonymous, £52 10s.; Anonymous, £10; total, £7,842 10s. In addition, Mr. William Cresswell Gray, J.P., has undertaken to add one-third of the amount raised, his individual contribution to be limited to £3,000.

